# Implementing administrative evidence based practices: lessons from the field in six local health departments across the United States

**DOI:** 10.1186/s12913-015-0891-3

**Published:** 2015-06-06

**Authors:** Kathleen Duggan, Kristelle Aisaka, Rachel G. Tabak, Carson Smith, Paul Erwin, Ross C. Brownson

**Affiliations:** Prevention Research Center, Brown School, Washington University, St. Louis, MO USA; Department of Public Health, University of Tennessee, Knoxville, TN USA; Division of Public Health Sciences and Alvin J. Siteman Cancer Center, Washington University, St. Louis School of Medicine, St. Louis, MO USA

## Abstract

**Background:**

Administrative evidence based practices (A-EBPs) are agency level structures and activities positively associated with performance measures (e.g., achieving core public health functions, carrying out evidence-based interventions). The objectives of this study were to examine the contextual conditions and explore differences in local health department (LHD) characteristics that influence the implementation of A-EBPs.

**Methods:**

Qualitative case studies were conducted based on data from 35 practitioners in six LHDs across the United States. The sample was chosen using an A-EBP score from our 2012 national survey and was linked to secondary data from the National Public Health Performance Standards Program. Three LHDs that scored high and three LHDs that scored low on both measures were selected as case study sites. The 37-question interview guide explored LHD use of an evidence based decision making process, including A-EBPs and evidence-based programs and policies. Each interview took 30–60 min. Standard qualitative methodology was used for data coding and analysis using NVivo software.

**Results:**

As might be expected, high-capacity LHDs were more likely to have strong leadership, partnerships, financial flexibility, workforce development activities, and an organizational culture supportive of evidence based decision making and implementation of A-EBPs. They were also more likely to describe having strong or important relationships with universities and other educational resources, increasing their access to resources and allowing them to more easily share knowledge and expertise.

**Conclusions:**

Differences between high- and low-capacity LHDs in A-EBP domains highlight the importance of investments in these areas and the potential those investments have to contribute to overall efficiency and performance. Further research may identify avenues to enhance resources in these domains to create an organizational culture supportive of A-EBPs.

**Electronic supplementary material:**

The online version of this article (doi:10.1186/s12913-015-0891-3) contains supplementary material, which is available to authorized users.

## Background

The tenets of evidence-based decision making (EBDM) in public health have been formally developed over the past 15 years in several countries. Evidence-based decision making is a process that involves the integration of the best available research evidence, practitioner expertise, and the characteristics, needs, and preferences of the community [1–9]. In local health departments (LHDs), this process includes the implementation of administrative evidence based practices (A-EBPs) [9]. Administrative evidence based practices are agency level structures and activities positively associated with performance measures (e.g., achieving core public health functions, carrying out evidence-based interventions) [10]. There are five broad domains of A-EBPs: leadership, workforce development, partnerships, financial processes, and organizational culture and climate (Table 1). These domains were previously developed from a literature review of evidence reviews that aimed to identify administrative practices of varying priority, determined by the length of time needed to modify them or the strength of their research support [10]. The five broad domains, and their 11 subdomains, are described as both high-priority and locally modifiable in a short to medium timeframe [10]. Use of A-EBPs in LHDs is important because these practices have been shown to be effective in boosting performance, contributing to accreditation efforts, and may ultimately lead to improved health of the population [9, 10]. In addition, the Public Health Accreditation Board requires that LHDs use and contribute to the evidence base, and likewise requires effective administrative practices – thus use of A-EBPs may fulfill multiple domains within the LHD accreditation process [11]. Since LHDs in the United States are using A-EBPs to varying degrees [12, 13], it is important to examine the contextual conditions that influence the implementation of A-EBPs. The purpose of this study, then, is to explore differences in LHD characteristics that may in part explain the differences in implementation of A-EBPs. In particular, this study will focus on contextual differences between high- and low-capacity LHDs, further defined below.

**Table 1:** Administrative evidence-based practices (A-EBPs)^a^ in local health departments

Domain	EBP	Description
Workforce development	Training	• In-service training in quality improvement or evidence-based decision making
		• Skills-based training (e.g., organization and systems change)
		• Training in communicating and collaborating with employees from multiple disciplines
		• Training aligned with essential services and usual job responsibilities
	Access to technical assistance	• Access and use of knowledge brokers^b^
		• Use of process improvement activities (e.g., accreditation, performance assessment)
		• Face-to-face meetings to share lessons, compare experiences, and provide updates
Leadership	Skills and background of leaders	• Leadership skill development
		• Leadership experience
		• Quality of leadership
		• Leadership influence
		• Manager competency to manage change
	Values and expectations of leaders	• Leadership support of quality improvement, national performance standards, evidence-based decision making, innovation, accreditation
		• Intend to hire well-educated, experienced staff including specialists (e.g., lab scientists, epidemiologists, environmental health professionals, financial systems experts)
	Participatory decision-making	• Broad participation among the management team
		• Leaders and middle managers seek and incorporate employee input
		• Non-hierarchical decision-making
Organizational climate & culture	Access & free flow of information	• Communication flow
		• Tailored messaging for evidence-based decision making
		• 360 degree employee performance reviews geared to evidence-based practices (with extensive feedback)
		• Ready access to high-quality information
	Support of innovation & new methods	• Leadership/management and employee training in evidence-based decision making that includes new methods
		• Employees perceiving that management supports innovation
		• Conscious creation of environments conducive to innovation
		• Organizational capacity to be in both business-as-usual state and state of exploration/innovation
	Learning orientation	• Shared employee perceptions that supervisors value learning and research evidence
		• Project management teams that encourage communication & collaboration
		• Presence of multidisciplinary, diverse management teams
Relationships & partnerships	Inter-organizational relationships	• Build and/or enhance partnerships with schools, hospitals, community organizations, social services, private businesses, universities, law enforcement
		• Cooperative agreements with state and/or local health departments quality improvement
	Vision & mission of partnerships	• Clear vision & aligned mission of partnerships
		• Capacity building over time among partners
Financial practices	Allocation & expenditure of resources	• Outcomes-based contracting
		• Resources allocated for quality improvement, evidence-based decision making, innovation, information access, training and implementation
		• Diverse funding sources

^a^Adapted from Brownson et al. [3]

^b^A knowledge broker is a masters-trained individual available for technical assistance

## Methods

### A mixed methods approach was utilized to expand upon quantitative findings from the *LEAD Public*

*Health National Survey* (LEAD survey) and further examine differences in LHD characteristics that influence the use of A-EBPs [12, 13]. Qualitative case studies were conducted among a select number of LHDs, in conjunction with a set of quantitative studies on the definition and use of A-EBPs in LHDs [9, 10, 12, 14–17]. The case study sample was selected using an A-EBP score from the LEAD survey (described elsewhere) [12] and secondary data from the National Public Health Performance Standards Program (NPHPSP). A set of A-EBP scores were derived from thirteen 7-point Likert scale questions from the LEAD survey and sum scores were then ranked into quartiles. Secondary data from the NPHPSP was linked to the LEAD survey; in concordance with NPHPSP scoring methodology, an overall performance score was computed as a simple average of the 10 Essential Public Health Services scores and then ranked into quartiles. “High-capacity” was defined as A-EBP scores in the top quartiles and “low-capacity” defined as scores in the bottom quartiles for both the LEAD survey and the NPHPSP.

Three LHDs that were in the top quartile and three from the bottom quartile of both measures were used as case study sites. The 6 sites were selected to provide a variation in geographic dispersion, governance structure and jurisdiction size. A goal of 6–8 interviews was used to achieve content saturation. Previous research shows that meaningful themes can be developed after 6 interviews and saturation is often present with 12 interviews [18]. All of the LHDs that were selected and approached agreed to participate in this research.

### Case study guide development

The interview guide (see Additional file 1) was developed based on previous literature [19–22], prior work by members of the research team (both researchers and practitioners) [23, 24], and research team input to explore LHD use of an EBDM process, including A-EBPs and evidence-based programs and policies. Evidence-based programs and policies include interventions, programs, and policies with evidence (based on published research) of improving health. Interview guide questions were developed to qualitatively supplement the data gaps from the quantitative national survey [12, 15]. The guide included the following topic areas: 1) biographical information; 2) awareness of the existence of an EBDM process; 3) administrative support for EBDM; 4) knowledge of the LHD accreditation process; 5) political climate and support for EBDM; 6) dissemination strategies that would further EBDM; and 7) key networks and partnerships to support EBDM.

### Cognitive response testing

In May 2013, the case study guide underwent cognitive response testing to elicit questions that were either unclear or potentially difficult to answer. Cognitive response testing is routinely used in refining questionnaires to improve the quality of data collection [25–28]. These 45–60 min phone interviews were conducted by the project manager with directors of LHDs in two states not selected as case study sites. The cognitive response testing sample (*n* = 6) was purposively selected by members of the research team. Upon verification of consent, all interviews were audio recorded, and field notes were taken during the interviews. Participants were instructed to provide feedback on questions lacking clarity and items that could be viewed as potentially difficult to answer. After the tester verbalized each question, the participant was allowed time to provide relevant feedback on each item. Information from these interviews was used to modify items and formulate the revised questionnaire for reliability testing. The final interview guide included 37 questions in the seven topic areas previously listed.

### Case study interviews

Interviews were conducted with 35 practitioners (including directors and assistant ant directors) from the six case study sites in June-July of 2013, with an average of five interviews per LHD. LHD directors and assistant directors selected a variety of practitioners/professional staff for interviews including program managers, clinic managers, and administrative or financial managers because these individuals were likely to be knowledgeable about the LHD’s EBDM practices. Each interview was conducted by two members of the research team and took 30–60 min, depending on the length of answers and knowledge of the practitioner. All participants provided informed consent before the interview began. This study received IRB approval from Washington University in St. Louis.

### Analysis

The interviews were tape recorded with the respondent’s permission and transcribed verbatim. Standard qualitative methodology was used for data coding using NVivo software. Four team members were trained on coding to ensure reliability among raters. A codebook was complied with inductive codes, and both inductive and deductive codes were used when coding the transcripts. Coders were assigned transcripts to code independently, after which the codebook was refined to capture new themes and subcategories. Updated codebooks were distributed after each coding session. Coding pairs systematically coded three interviews using NVivo noting any discrepancies and alternate coding. Once these transcripts were coded and the codebook refined, inter-rater reliability was evaluated using NVivo with a final percent agreement among coders of 98 %. Data from each LHD was summarized and combined into high-capacity LHD and low-capacity LHD categories. Node reports were generated to explore common themes in the high-capacity and low-capacity LHDs and then summarized into thematic reports for each of the five A-EBP domains.

## Results

Of the three LHDs categorized as high-capacity, two had local governance and one had shared governance between the state and LHD. One LHD was in each of these three jurisdiction sizes: 500,000+; 100,000–499,999; and 25,000–49,999. Two of these LHDs were in the Midwest census region and one in the South census region. The three LHDs categorized as low-capacity had two state-governed health departments and one with shared governance. Two of them had population jurisdiction sizes between 50,000–99,999 persons, and one between 25,000–49,999 persons. There was one LHD in each of the census regions of the South, Northeast, and West.

From the thematic reports, the similarities and differences of high-capacity and low-capacity LHDs were compared across the five A-EBP domains and organized into an A-EBP table (Table 2). Based on the A-EBP table, specific themes and patterns were identified and explored. The domain of relationships and partnerships was very similar for both high- and low-capacity LHDs—both groups reported that they value partnerships and often share expertise and staff time with their partners. The only difference that appeared was specific to internal relationships within the LHD. Consequently, we have limited the discussion of partnerships to the differences in internal relationships that have been grouped under organizational culture and climate. The domains of workforce development, leadership, and organizational climate and culture had the most dramatic differences between high and low capacity LHDs.

**Table 2:** Comparison of high and low capacity local health departments (LHDs) by A-EBP domain

AEBP	High	Low	Both
Workforce development			
Training	- Budget line item for continuing education	- No financial support to go to trainings	- Recognize the need for trainings
	- Try to send staff to all state and some national conferences	- Very few if any attended	- More are needed
	- Use time during all staff meetings to conduct trainings		- Potential areas of focus: accreditation, webinars (as opposed to in-person training), specific topic-related conferences
Leadership			
Skills & background of leaders^a^	- Physician/MPH/PhD	- Masters in Management	
	- Bachelors in SW, MSW, completing MPH	- Bachelors in nursing, certificate of grad study in fundamentals of public health	
	- Bach in science and education		
Values & expectations of leaders	- 100 % supportive of use of EBPs	- Directors mostly supportive of EBPs	- Supportive of EBPs
	- Expect LHD to grow and change including use of EBPs	- Not all upper management were supportive of EBPs	- Know EBPs should be used
	- Feel it is their job to provide direction and training for their staff in EBPs	- Poor communication of EBPs and expectations	- Want to provide quality service for the clients
Participatory decision-making	- Decisions often made by consensus	- State makes many decisions	- Subject matter experts at the LHD consulted
	- Ideas come up from staff to management and tested	- Decisions mostly made by upper level management team	- State and regional HDs give directives
	- All staff meetings once a month to gather and distribute ideas	- Director makes decisions after evaluating staff ability and capacity for programs	- Involve community members and stakeholders
Organizational climate & culture			
Access and free flow of information	- University libraries	- Very little access to online or paper journals	- Internet access
	- Attendance at conferences, in-person and online trainings		- Information from state office
	- Some academic journal subscriptions through LHD		- National Association of County and City Health Officials
Support of innovation & new methods	- Supports and encourages new ideas	- Many people in the LHD are adverse to change	- Would like staff to be open to change more
	- Uses QI to explore things that can be changed to improve LHD	- No flexibility to try anything new because many priorities are mandated by the state	
	- Hires employees that are willing to change with the LHD	- New ideas are not well-received	
	- “Global” instead of “Silo” approach to programs		
Learning orientation	- Send staff to conferences and/or conduct trainings at the LHD show support of learning	- Do not necessarily emphasize collaboration, especially multidisciplinary	- Would like to send staff to more trainings and conferences but can’t due to lack of budget
	- Many staff go back for MPH while working		
	- More multidisciplinary collaboration within the LHD		
Relationships & partnerships			
Interorganizational relationships	- Some sharing of funding between partners including grants from the community	- Funding is only shared within the department	- Feel partners are essential to work of the health department
	- Share facilities	- Looking to community assessment to bring LHD and community partners closer	- Share staff time and resources with partners
	- Community partners have been involved in trainings		
Vision and mission of partnerships	- Seems collaborative	- Many partnerships seems to be one working for the other instead of collaborative	- Come together for the good of the citizens
Financial			
Allocation and expenditure of resources	- State department provides funds to the LHD to prepare for accreditation	- No funding that is not already earmarked for specific programs	- Lack of finances is major roadblock to implementing EBPs and EBDM Process
	- Line item in the budget for trainings and conferences	- Positions have been cut due to budget cuts	- Lack of funding to pay high salaries can lead to hiring of staff that has less experience and/or less education
	- Several staff felt their LHD has the financial stability needed	- Financial situation makes even mandated programs difficult to implement	

^a^Self-reported by LHD leaders

### Workforce development

High-capacity LHDs often mentioned training as an important aspect of their work; for example, employees mentioned opportunities to attend state and national conferences. Two of the high-capacity LHDs also mentioned using staff meetings to have on-site trainings about the EBDM process, accreditation documentation, or continuous quality improvement. One participant from a high-capacity LHD described:

“there is a line item for education or continuing education [for] our staff. So if people need a certain type of training […] we have that and we provide that to our employees to make sure they’re all certified.”

Staff at low-capacity LHDs expressed the desire to attend trainings and conferences, but said funding constraints and travel restrictions do not allow them to attend. One participant from a low-capacity LHD mentioned:

“We can go to [one specific] conference, but anything else, we do on our own. It hasn’t always been like that, but it has the last several years.”

### Leadership

Leadership encompasses values and expectations of leaders as well as participatory decision making at the LHD. Leadership at both sets of LHDs expressed the knowledge that it is desirable to use evidence-based programs and policies, but employees at the high-capacity LHDs more often noted behaviors of the leaders as being intentional for the purpose of promoting the use of EBPs. Leaders at the high-capacity LHDs were more likely to be fully supportive of EBPs, to actively provide direction and training for staff in EBPs, and to convey the expectation that the LHD would continuously grow and change. When asked about decision making, staff at high-capacity LHDs mentioned group decision making, ideas generated by non-managerial staff, and all-staff meeting time used for the purpose of gathering and distributing ideas. One participant from a high-capacity LHD commented,

“It’s important enough to administration that they have the time to do the research and to attend the academic classes or the trainings and things that they need to keep us current on best practices.”

Staff at low-capacity LHDs, in contrast, had mixed feelings about leaders’ support for EBPs; one mentioned that

“I’ve found it from my director, but not necessarily some of the other leaders.”

Additionally, lack of communication regarding expectations for using EBPs, as well as how and when to use them, emerged as a theme in low-capacity LHDs. Decision making at the low-capacity LHDs was often done by the management team or director. However, many decisions were said to be made at the state or regional level without input from anyone at the LHD.

### Organizational climate and culture

Access to information, support of innovation, and learning orientation are part of organizational culture and climate. Overall, staff at high-capacity LHDs had better resources to access more information; they described access to university libraries, academic journal subscriptions, or trainings to get information. In contrast, staff at the low-capacity LHDs had little access to online or printed paper journals. Regarding support of innovation, the culture at high-capacity LHDs was described as encouraging to new ideas and open to changes that would improve the overall LHD. One participant from a high-capacity LHD commented that their LHD encourages employees to:

“Always try to improve things, try new things, that’s fine. And if you make a mistake doing that, you’re not going to be fired for that, you’re not going to be reprimanded for that; you’re going to try something new, something different.”

They also mentioned more collaboration within their LHD; one participant described that:

“one of the things that we have done an exceptional job at doing is breaking down silos [….] we have more of a global approach, an open approach, that allows us to get things done and get things done fairly efficiently.”

Low-capacity LHDs, on the other hand, were described as having cultures that were averse to change and without flexibility due to state mandated programs. On the topic of new ideas and changes, one participant from a low-capacity LHD described:

“There are some up and coming individuals who have different ideas and different ways of doing things, but I can’t say at this point that it’s extremely well-received.”

Related to the A-EBP domain of relationships and partnerships, low-capacity LHDs overall were also less likely to highlight multidisciplinary relationships, instead only mentioning collaboration with specific individuals or directors within their departments.

### Financial practices

Differences between high- and low-capacity LHDs were evident in the domain of financial practices as well. This was most apparent when looking at the reported flexibility of funding within the department. Low-capacity LHDs had little to no flexible funding and reported they can only implement state mandated programs. Some of these LHDs were experiencing staffing shortages and felt they were unable to implement programs fully due to this shortage and to budget constraints. One participant from a low-capacity LHD mentioned:

“Because we do not have latitude in how we spend money, I think … it probably impedes our ability to think about solutions to problems that could be affected had we been able to obtain and sustain [funding for programs].”

High-capacity LHDs also reported that they would like more funding, but had some flexible funding to use on the programs they thought were best for their LHD. They also seemed to be more optimistic about meeting goals despite financial difficulties. One participant from a high-capacity LHD pointed out:

“There’s always a gap [between what we would like to have and what’s available]. As long as we’re on board and we recognize those challenges, we do the best we can to meet all those goals.”

## Discussion

High-capacity LHDs were more likely to have the leadership, organizational culture, and financial capacity to support workforce development activities, through sending staff to trainings and conferences and/or using meetings and training opportunities. In addition, high-capacity LHDs mentioned that more supportive, communicative leadership goes farther in building a department that is resilient to setbacks or problems that may arise. More specifically, they seemed to have more accepting, supportive cultures that value innovation and encourage collaborative communication compared to low-capacity LHDs. High-capacity LHDs were also more likely to mention working with a wider range of staff across their LHD, instead of particular individuals or staff within their own work unit. Financial constraints were a huge barrier for both high- and low-capacity LHDs; however, high-capacity LHDs seemed more flexible and open to making things work. Low-capacity LHDs were more likely to describe limited or insufficient funding as an insurmountable obstacle. Lastly, high-capacity LHDs were more likely to describe having strong or important relationships with universities and other educational resources, which increases their access to resources and allows them to more easily share knowledge and expertise.

### Relationship to findings from previous research

Workforce development emphasizes the importance of focusing on the core competencies for public health professionals, incorporating them into LHD missions, visions, and goals. Providing trainings for employees in quality improvement or EBDM, leadership skills, multidisciplinary approaches, and other areas increases growth and learning, enhancing the capacity and reach of a LHD [17, 29]. Workforce development has been linked to better performance, which ultimately leads to better community health outcomes [17, 29].

Enhancing leadership includes having competent leaders that can effectively communicate missions and visions, and are knowledgeable about and supportive of quality improvement, accreditation, national performance standards, EBDM, participatory decision-making and non-hierarchical collaboration [30]. It may also involve having leaders with sufficient amounts of skill, experience, and influence, as well as having a competent workforce that is able to take on leadership positions within the LHD. Leadership is especially important in that it is the driving factor behind other A-EBPs—leaders who understand the importance of EBDM are more likely to prioritize workforce development and emphasize a specific kind of organizational culture, effecting further growth within their LHD [31].

An effective organizational culture has a learning orientation that encourages new thinking and adapting to new environmental conditions, rather than just doing what has been done in the past. It also includes support and training that incorporates innovation and new methods, valuing diversity and unique perspectives [17]. This is made possible through access to high-quality information and feedback from leaders about employee performance. Additionally, prior research suggests that the introduction and use of specific resources and tools across LHDs should be prioritized as an effective organizational strategy [32].

Allocating resources and actively promoting the use of A-EBPs (e.g., supporting quality improvement, EBDM, training) can improve health department performance and community health overall [29]. Easily accessible tools and resources can reduce time and cost barriers to EBDM within LHDs, improving both effectiveness and efficiency [32]. Additionally, obtaining funding from multiple, diverse places gives LHDs greater flexibility in spending and lessens dependence on only a few core sources [33].

Finally, building and enhancing relationships with multidisciplinary partners and being able to identify and clarify a shared vision helps to increase rates of change, sustainability, and capacity building over time [17, 29].

### Implications

Low-capacity LHDs may benefit from identifying more creative, cost-efficient strategies for enhancing workforce development. Research suggests that incorporating meetings and trainings that are more interactive and problem-specific, as well as emphasizing autonomy, prior knowledge, and relevancy, will be more effective in developing a more educated, competent workforce [34]. Workforce development training that emphasizes leadership skills may also be beneficial, as leaders can have a tremendous influence on other areas of the LHD and overall productivity, especially in terms of what kind of supportive communication and action takes place [35].

Low-capacity LHDs could benefit from leaders who emphasize and value A-EBPs through communication, training opportunities, funding, and other means. Increased leadership support across various levels and departments within the LHD could facilitate change in organizational culture and climate, helping staff to be more comfortable with EBPs and the process of EBDM [36]. Also, high-capacity LHDs in this sample have leadership who value innovation and create a culture that supports risk taking by encouraging staff to try new ideas. If a new idea doesn’t work, they learn from it and try something else. This creates an environment that is supportive of change and is not of afraid of failure.

Lastly, research has suggested that partnerships between academia and LHDs are critical for addressing public health needs and successfully improving a community’s overall health and well-being [37]. Thus, exploring avenues to enhance collaboration and resource exchange between universities and LHDs may help to lessen the gap between low- and high capacity LHDs.

### Limitations

The main limitations of this study are that the data are self-reported and the sample size was small, thus limiting generalizability. In addition, practitioners interviewed were selected by the director and this could introduce selection bias. Finally, the LHDs in high and low capacity categories differed in size, governance structure, and geographic region – all of which may independently impact or influence performance capacity. Specifically, the group of high-capacity LHDs chosen by our ranking method had larger jurisdiction sizes in comparison to the group of low-capacity LHDs, which may have factored into their ability to address A-EBPs. A more in-depth exploration of how high- and low-capacity LHD performance differs based on size, governance structure and geographic region is an area needing further study.

## Conclusion

Differences between high- and low-capacity LHDs in A-EBP domains highlight the importance of investments in these areas and the potential those investments have to contribute to overall LHD efficiency and performance. Low-cost resources exist for low-capacity LHDs to better their performance, including free A-EBP issue briefs that give background information and specific resources related to each of the 5 A-EBP domains, a resource toolkit about A- EBPs that lists online resources available to LHDs [38], training courses to improve EBDM [39], and the National Association of City and County Health Officials’ EBDM resource site for LHD practitioners [40]. Additionally, low-capacity LHDs might consider seeking higher-capacity LHD mentors or partners, as well as increasing cross-jurisdictional sharing of resources. Enhancing access to resources and technical assistance to improve A-EBP use in LHDs should be explored further. Also, enhancing leadership skills to foster a more flexible environment supportive of innovation may enhance capacity in LHDs. Lastly, policy makers and researchers should strive to offer easily accessible trainings to LHDs. Investments in A-EBPs have the potential to increase readiness for LHD accreditation, improve overall performance, and improve health outcomes in communities.

## Additional file

Additional file 1:
**Case study Interview Guide.**
