# Supplementary material for: Implementing administrative evidence based practices: lessons from the field in six local health departments across the United States
Source: BMC Health Serv Res. 2015 Jun 6;15:221. doi: 10.1186/s12913-015-0891-3 (PMC4457307; doi:10.1186/s12913-015-0891-3)
Supplement: Additional file 1: — Case study Interview Guide. [file 12913_2015_891_MOESM1_ESM.pdf]

Additional file 1 – Case study Interview Guide

<u>Objectives</u> (if asked to describe the study/project in more detail): [these only get stated if someone asks for more information]

1. *Understand Contextual conditions in the implementation of evidence based programs/policies in local health departments, using primarily qualitative methods.*
2. *Explore several key domains related to program and policy implementation in your health department:*
    a. *Biographical information;*
    b. *Awareness of the existence of evidence-based programs/policies*
    c. *Administrative support for evidence based programs/policies*
    d. *Knowledge of the Public Health Accreditation Board (PHAB) accreditation process and of your state accreditation process (if applicable)*
    e. *Political climate and support for evidence based programs/policies*
    f. *Key networks and partnerships to support evidence-based decision making*

Thank you for agreeing to participate in this interview. As we mentioned in the email your responses today are confidential and will not be used in any way to identify you or your health department.

Before we begin, just to let you know there are no right or wrong answers, and you might not know the answer to all the questions, and that's fine, feel free to let us know and we will skip that question.

<u>Part 1: Awareness of the existence of evidence-based processes</u>

We will begin with a few questions about your agency's **<u>use</u>** of an evidence-based process to plan and carry out public health services, programs and policies. For the purposes of this interview, we define the evidence-based process as one that uses the following approaches:

- Making decisions based on the best available scientific evidence;
- Applying program planning and quality improvement frameworks;
- Engaging the community in assessment and decision making; and
- Conducting sound evaluation.

We will also be talking about **Evidence-based interventions,** which are programs and policies with evidence (based on published research) of improving health.

So that you can refer to them throughout the interview, these definitions can also be found on your information sheet.

1.1 Which of the following best describes the extent to which the *Guide to Community Preventive Services* (*Community Guide* or www.thecommunityguide.org) has been used to support or enhance decision making in your LHD over the past 12 months?
❑ LHD staff have not used the *Community Guide*
❑ LHD staff in a few programmatic areas have used the *Community Guide*
❑ LHD staff in many programmatic areas have used the *Community Guide*
❑ The *Community Guide* is used consistently by all relevant programmatic areas
❑ Do not know the extent of use of *Community Guide* within LHD

1.2 Are you aware of any evidence-based interventions conducted in your jurisdiction that you consider a good example? **Evidence-based interventions,** are programs and policies with evidence (based on published research) of improving health. This definition can also be found on your information sheet.

Probe:
If YES:
Describe the program(s)?
Why do you think this is a good program?
What is the target population?
For how long the program has been conducted? What is your role in this program?
Can you think of any examples more specifically related to chronic disease prevention and health promotion that have been supported by your department?

If Participant does not know about EBDM or EBPP you can ask "If you were going to design a program how would you go about doing that? Where would you get information?

1.3 Does your department implement program planning and quality improvement processes? (For example: Balanced Scorecard, Baldrige Performance Excellence Criteria (or state version), Lean, Plan-Do-Check-Act or Plan-Do-Study-Act, Six Sigma)
Probe:
If Yes:
Please describe the processes.
How do you think these processes help your organization?
What resources are allocated to allow for the implementation of these processes?
What else about your department facilitates using such processes?
If No:
Please tell me what processes you are aware of or those being used in other departments.
How do you think a program planning and quality improvement processes might improve your department?
What resources would allow you to implement such processes?
What else might facilitate implementing these processes?

<u>Part 2: Administrative support for evidence based programs/policies</u>

The next questions ask about your agency's **<u>support</u>** of the use of an evidence-based process to plan and carry out public health services, programs, and policies.

2.1 How would you describe the culture of your department as it relates to implementing evidence based processes?
Probe:
How much support do you feel the department provides for the processes necessary for utilizing evidence based programs/policies?

2.2 What kind of access do you have to current research evidence for evidence based processes?
(e.g. Academic journals, Reports to funders, Press releases, Newsletters, Policy briefs, Email alerts, Targeted mailings, Academic conferences, Seminars or workshops (phone, webinars, or in-person), Face-to-face meetings with stakeholders, Media interviews, CD-ROMs, Social Media (Facebook, Twitter), Professional associations)
How relevant is this research to the community you serve?

2.3 Do leaders in your institution provide support for an evidence based process?
Probe:

What type of support?
For whom the support is provided?
How often?

2.4 Do you find administrative support (i.e. personnel, financial, access to information) for an evidence based process in your agency when you need it?
Probe:
If Yes:
How frequently is this support necessary?
If NOT:
What are the reasons for not getting the support you need?

2.5 When thinking about an evidence based process, what is the type of support that you need most often?
2.8 If you were able to change one thing related to the culture of your department and its support of an evidence based process, what would it be?

2.6 In your experience in hiring employees, what qualifications do you seek in employees to be sure they can carry out activities in evidence based processes?
Probe:
What are the barriers to hiring these individuals?
How important are credentials (such as public health degrees) considered?
How important is past work experience in public health considered?
Why are these factors (un)important?
What other factors are considered as part of the hiring process?

2.7 Have you ever hired a person specifically for the purpose of developing or using evidence based processes?
Probe:
If so, what have you learned from this process?
What did you look for in this candidate?

Part 3: Political climate and support for evidence based interventions

The next questions ask about the political climate and support for evidence based interventions. Please note that up till this point in the interview, we had been discussing evidence based processes. We are now discussing **Evidence-based interventions,** which are programs and policies with evidence (based on published research) of improving health. This definition can also be found on your information sheet.

3.1 Think about an evidence-based intervention you described earlier. Can you think of things that helped you to implement these interventions?
Probes:
Funds
Adequate staff
Support from administrators/mangers within your health department
Support from elected officials
Existence of/partnerships with coalitions
Mandates

Training
Experience
Knowledge
If you do not support any evidence based interventions, what would help you implement such interventions?

3.2 Can you think of any roadblocks to implementing evidence-based interventions?
Probes:
If Yes:
What are the barriers?
Such as familiarity/knowledge about EBIs
Support within your department
Financial/staff resources
Cost effectiveness information
Training
Experience
Knowledge
If no:
What is it about your department that minimizes road blocks?

3.3 When you think about your ability to sustain an evidence-based interventions, what are the contributing factors?
Probes:
Funds
Engaged partners
Adequate staff
Evaluation capacity

Part 4: Networks and partnerships to support evidence-based decision making

The next questions ask about networks and partnerships to support evidence-based processes. Collaborative **partnerships** (people and organizations from multiple sectors working together in common purpose) are a prominent strategy for community health improvement. This definition can also be found on your information sheet.

4.1 Does your department collaborate with partners in health the sector (i.e. medical providers, hospitals, Federally Qualified Health Centers) and/or outside the health sector (e.g. police departments, the United Way, parks & recreation departments, civic groups, chamber of commerce, faith-based organizations, schools, businesses, academic partners)?
If YES:
Please describe?
What has led to your most successful partnerships?
How were these relationships forged?
How do these partnerships influence your departments ability it implement evidence based interventions?
How long has the collaboration existed?
If No:
With whom would you like to partner?

4.2 Thinking about the partnerships you just described, who are the top 5 organizations (outside of your LHD) with whom you most frequently collaborate with on health department activities?

4.3 Who are the top 5 colleagues within your LHD that you most frequently collaborate with on health department activities?

4.4 Do you think that collaboration and network are critical for your department to achieve its goals and mission? Why or why not?

4.5 What are the roadblocks for increasing collaboration?

4.6 In your partnerships, what type of resources do you share?

Probe:

Staff
Funds
Expertise

4.6.a. How do you feel this sharing supports collaborative work?

Part 5: Dissemination strategies that would further evidence based programs

The next set of questions asks about dissemination strategies related to evidence based interventions. We believe dissemination comprises an active and planned process rather than a passive approach to spreading evidence based interventions.

5. How does your health department disseminate evidence based programs and policies?

5.1. When you want to make decisions or influence decisions related to evidence based interventions in your department what are the most useful approaches to decision making?

5.1.a. How often do you use evidence based interventions?

5.2 What avenues allow you to learn about the current findings in evidence based interventions (e.g. peers from other LHDs, academic journals, conferences, government reports, social media, Reports to funders, Press releases, Newsletters, Policy briefs, Email alerts, Targeted mailings, Academic conferences, Seminars or workshops (phone, webinars, or in-person), Face-to-face meetings with stakeholders, Media interviews, CD-ROMs, , Professional associations)

5.2.a. How do you access these methods?

5.3. If you were going to enhance the use and dissemination of evidence based programs, what would need to happen?

Part 6: knowledge of the accreditation process for local and state health departments?

The next questions ask about your knowledge of the accreditation process for local and state health departments

6.1 What types of accreditation are you aware of?

If PHAB is not mentioned….

6.1.a. Are you aware of the PHAB accreditation process?

If YES:

6.1.b. How ready do you feel your department is to participate in the National Voluntary accreditation process for local and state health departments?

Probe:

If high:

What do you think has allowed your department to be ready?

If Low:

What would help your department be more ready?

6.2 In the PHAB Domain Standards, measure 10 states "*Identify and use applicable evidence-based and/or promising practices when implementing new or revised processes, programs and/or interventions"* More information about this standard is available on the information sheet. Do you feel your health department is currently able to meet this measure for PHAB?

We are almost done with the interview. I have just a few questions about your background.

Part 7: Biographical Information and Experience

7.1 What is your gender [ask only if needed]?

7.2 What is your age?

- ❑ 20-29
- ❑ 30-39
- ❑ 40-49
- ❑ 50-59
- ❑ 60-69
- ❑ 70-79

7.3 Do you hold a degree (e.g. BSc, MPH or PhD) or credentials in Public Health?

Probe: If YES, what degree? When did you receive?

7.4 How do you best describe your job/current position?

- ❑ Public health manager
- ❑ Public health physician
- ❑ Environmental health worker
- ❑ Health educator
- ❑ Epidemiologist
- ❑ Nutritionist
- ❑ Public health nurse
- ❑ Public health informatics specialist

❑ Public information specialist
❑ Behavioral health professional
❑ Emergency preparedness staff
❑ Administrative or clerical personnel
❑ Administrator/Director
❑ Other (please specify) ______________________

7.5 How long have you been in your current position?

7.6 How long have you been with this agency or organization?

7.7 How long have you worked in public health overall?

7.7 Please describe the primary responsibilities of your position?

Closing

Thank you very much for taking the time to talk with us about this study. We'd like the opportunity to conduct this interview with other colleagues within your Health Department. Can you help us identify others who might be willing to participate?

Collect name, title, email, and phone number

We'd also like to follow up with you to get a bit of additional information about the network of people you work with, within your health department. This would be done via email, asking all of your LHD colleagues the following two questions:

(1) *Name the five colleagues at (LHD name) you communicate with the most on health department activities:*

(2) *Name the five organizations outside the LHD you communicate with the most on health department activities:*

If you have any questions please feel free to contact the project manager Katie Duggan at 314 935-0125 or kduggan@wustl.edu
